# Dr. Henry O. Marcy

**Published:** 1890-12

**Authors:** 


*Letter from Dr. Henry O. Marcy — Apostoli's Latest Conclusions on the Use of Electricity in Uterine Myomas — Abstract of His Paper at the Berlin Congress.*

The following letter from Dr. Marcy is published as a preface to the Apostoli treatment, for the information and benefit of our readers, and as a fitting introduction to the subject:

*Editors Buffalo Medical and Surgical Journal:*

Since the Apostoli method of treatment of uterine myoma by electricity engages at the present a prominent share of attention by the readers of the Journal, I send you a translation of Apostoli's conclusions, as given at the late Berlin Congress, which I have not seen published, and which was given me personally by the author. Conclusions as to results may differ, but I am glad to bear testimony to the earnest, impartial, conscientious spirit of scientific investigation which pervades all of Apostoli's work. I was too incredulous to even give the method a trial until after his visit to America three years ago. Since then I have made about 1,000 applications of the constant galvanic current in cases of uterine myomata, with varying results; with one exception, I am sure without harm, and this doubtful if referable to electricity. I am convinced the cases should be carefully selected, and fortunately the class where the greatest good is likely to follow is that least amenable to surgical treatment, the growths filling the pelvic cavity. The intrapolar current must traverse the growth in order to modify its nutrition. The menorrhagic cases are almost without exception benefited.

HENRY O. MARCY.

116 Boylston Street, Boston, Nov. 1, 1890.

Dr. Apostoli gave a lecture at the Congress, of which the following are his conclusions :

1. The constant galvanic current finds its principal indication in gynecology in endometritis and fibroma, regulating the circulation and controlling amenorrhea, dysmenorrhea, and metrorrhagia. It is of value in stopping the evolution of benign neoplasms, and aids in the resorption of the peri-uterine exudates. It exercises a resolutive action very salutary in many of the peri-uterine phlegmasias, and in certain ovari-salpingites, catarrhal ; but it is not efficacious and even harmful in high dosage, especially if the intrauterine pole is negative, in phlegmasia of the annexes.

The suffering caused by the current will increase with the inflammatory condition of the annexes, and ought to serve as a valuable means of diagnosis in determining the existence and the nature of the peri-uterine liquid collection, hematic or suppurative, which may be unknown or simply suspected, and a resort to, if suppurative, prompt surgical interference should be advised.

2. The effects of the constant galvanic current are polar, or inter-polar ; the inter-polar, trophic and dynamic, which increases as the square of the intensity increases and augments the polar action. This utilizes at first each pole in a different way, which Apostoli has called the caloric action, developed afterward by the passage of the current, in order to increase the interstitial circulation, and at length the antiseptic action of the positive pole, of which Apostoli and Laguerriére have recently given experimental demonstration.

3. The high galvanic applications, used in a variable manner, above fifty milliampères, according to the tolerance of the patient and the multiple clinical indications, form the fundamental basis of the Apostoli method, and find justification, firstly, in the utilization of circulatory drainage, the direct result of the caloric action, due to the resistance of the passage of the current and proportional to the square of the intensity.

Secondly, in the antiseptic, or microbic action which increases with the intensity of the current.

Thirdly, in the rapidity and efficacy of the effects produced, which are proportional to the square of the electric energy, after a formula, analogous to that of the measure of the energy of other natural forces.

Fourthly, in the generalization of the method to the rebellious cases, (fibroma and sub-peritoneal endometritis, fungous growths, etc.,) and in young women.

Fifthly, in the removal of the deposits resulting from inflammation, which, other things being equal, will be lessened in danger in proportion to the intensity of the galvanic current.

The vaginal application of the galvanic current, which is the method selected by Monsieur Chéron for fibromas only, and since applied by A. Martin, Bracket, Meniére, Carpenter, Mundé and others, gives results very inferior to the intra-uterine applications which should remain the method of choice, because it utilizes, above all, the maximums of the constant current, and of its energy. It also utilizes the antiseptic power of the positive pole, which is wholly local, and which is lost in the inter-polar circuit, or at the negative pole. It places often in contribution the derivative or caustic action of the intra-uterine application, treating thus at the same time either simple endometritis or endometritis complicated so often with fibroma and peri-uterine phlegmasia, assuring thus a more rapid, complete and permanent cure. It permits better than the vaginal application the minimizing of pain, and renders more tolerable the use of high dosage, and it assures, finally, a greater efficacy by rendering possible an increase of intensity applied, and of the bloody irrigation which it induces.

The vaginal galvano-punctures, made at some millimeters of depth (from 2-5) by the aid of a filiformed trocar, isolated in its whole extent, except at the point, forms the complement, often very salutary, of the intra-uterine therapeutics created by Apostoli, in localizing better the galvanic action, and in rendering more efficacious, in certain cases, the application of small or medium doses.

The harmlessness of the intra-uterine therapeutics is affirmed at first by the parallel harmlessness of the chemical, or bloody methods of the intra-uterine curetting, and especially by the statistical figures gathered in the entire world, and Apostoli's own résumé, which is as follows:

From July, 1882, to July, 1890, he made 11,495 galvanic applications, which are divided thus: 8,177 galvano-caustic intra-uterine positive; 2,486 intra-uterine negative galvano-caustic; 222 vaginal positive galvano-punctures; 614 vaginal negative galvano-punctures. He treated 912 patients, comprising 531 fibromas, 133 simple endometritis, 248 endometritis, complicated with peri-uterine phlegmasia, and these are divided thus:

*Clinical.*— 313 fibromas; 70 simple endometritis; 163 complicated endometritis.

*Indoor Patients.*—218 fibromas; 63 simple endometritis; 85 complicated endometritis.

Three patients died, chargeable to operative faults, viz.: two galvano-punctures, of which one was a sub-peritoneal fibroid, the other, an ovaro-salpingitis; and one, a caustic galvano-puncture, which was taken for a fibroid. He has observed thirty cases of pregnancy supervening after intra-uterine galvanic applications.

